# A Curious Pocket-Piece

**Published:** 1899-03

**Authors:** 


### A CURIOUS POCKET-PIECE.

[AUTHOR'S ABSTRACT.]

In the *New York Medical Journal* of February 4, 1899, Dr. William S. Gottheil describes a case in which a woman carried a piece of her own skull in her pocket for years "for good luck." She applied for treatment for a different affection, and it was discovered incidentally that a syphilitic periositis had begun again around the scar left by the ulceration from which her piece of bone had come twelve years before. As in the present case, she had not at that time attached sufficient importance to the matter to consult a physician about it. The sequestrum, of which she was quite proud, was an ovoid piece of bone measuring 2¼x2 inches, and was composed of two adjacent portions of the two parietal bones, the sagittal suture in the middle shewing beautifully. Its upper convex surface shewed the outer table of the skull intact. The under concave surface was composed mostly of cancellous tissue; but all along the middle line, at the suture, the inner table was present, shewing that at that place the entire thickness of the skull had been lost.

Apart from its curiosity, the case is of interest as shewing the very extensive destruction of important organs that can take place in syphilis without systemic reaction or much personal inconvenience. The entire thickness of the skull had been destroyed, and the meninges necessarily exposed; yet the inflammation had not spread to those membranes, and the patient had hardly considered herself sick.

